# Impact of a complex intervention in primary care for patients with palliative care needs in their healthcare utilization: a before-after study

**DOI:** 10.1017/S1463423625100777

**Published:** 2026-01-30

**Authors:** Carlos Seiça Cardoso, Filipe Prazeres, Cátia Nunes, Pedro Simões, Carolina Aires, Patricia Rita, Joana Penetra, Paulo Lopes, Sara Alcobia, Barbara Gomes

**Affiliations:** 1 Faculty of Medicine, University of Coimbra, Coimbra, Portugal; 2 Faculty of Health Sciences, University of Beira Interior, Covilhã, Portugal; 3 Family Health Unit Beira Ria, Gafanha da Nazaré, Portugal; 4 CINTESIS@RISE, MEDCIDS, Faculty of Medicine of the University of Porto, Porto, Portugal; 5 Family Health Unit Condeixa, Portugal; 6 Personalized Health Care Unit Fundão, Fundão, Portugal; 7 Family Health Unit São Martinho de Pombal, Portugal; 8 Personalized Health Care Unit Castanheira de Pera, Portugal; 9 Family Health Unit Topázio, Portugal; 10 Family Health Unit Rainha Santa Isabel, Portugal; 11 Family Health Unit Penacova, Portugal; 12 King’s College London, Cicely Saunders Institute of Palliative Care, Policy and Rehabilitation, London, United Kingdom

**Keywords:** Facilities and services utilization, palliative care, primary healthcare

## Abstract

**Objectives:**

Palliative care development in primary care is crucial worldwide. This study reports on the secondary outcomes of a study that evaluated whether a complex intervention in primary care for patients with palliative care needs impacted their healthcare utilization.

**Methods:**

A before-after study was conducted with family physicians and patients with palliative care needs. Physicians received palliative care training and implemented a new primary palliative care consultation model. Healthcare utilization in the 12 weeks before, during, and after the intervention was compared.

**Results:**

We analysed healthcare utilization for 33 patients with advanced disease. Pre-intervention, there were high rates of no medical consultations, emergency visits, hospital admissions, and outpatient referrals (84.8%, 75.8%, 81.8%, and 84.8%, respectively). Despite slight reductions during and after the intervention, the differences were not statistically significant.

**Conclusions:**

The reduction in healthcare utilization was not statistically significant, but the data inform sample size calculations for future economic analyses.

**Trial registration:**

ClinicalTrials.gov ID – NCT05244590. Registration: 14th February 2022.

## Background

The number of patients with chronic diseases has been increasing, especially due to the aging population, making palliative care a clinical and moral imperative (Prince *et al.*, 2015; Sleeman *et al.*, 2019). It is known that the majority of patients with palliative care needs do not have access to specialist palliative care (Tobin *et al.*, 2022), so healthcare systems must explore the provision of generalist palliative care. This makes primary palliative care a priority.

The evidence on the effectiveness of interventions targeting patients with palliative care needs in primary care is scarce, but it is known that when the family physician is involved, the provision of palliative care seems to improve, with benefits for patients (McWhinney and Stewart, 1994; Mitchell, 2002). Evaluations of specialist home-based palliative care services have shown the potential for cost savings elsewhere in the healthcare system, especially in terms of hospital expenses (Ij, 2013). It is also known that the clinical follow-up offered by primary care providers, including family physicians, to patients with chronic conditions leads to reduced mortality rates, decreased healthcare utilization (fewer emergency department (ED) visits, hospitalizations and readmissions), and lower healthcare costs (Kolber *et al.*, 2023). This may also apply to patients with palliative care needs. We therefore aimed to evaluate whether a complex intervention involving training in palliative care and a new consultation model implemented by family physicians would have an impact on the utilization of healthcare services by patients with palliative care needs.

## Methods

We conducted a before-after study involving family physicians working in a health region in Portugal and patients with palliative care needs from their lists of patients. The study was approved by the ethics committees of the Faculty of Medicine, University of Coimbra (CE-055/2021) and of the regional health authority (ARSC-15/2021) and registered in ClinicalTrials.gov (NCT05244590) (Seiça Cardoso, 2023). Each patient was required to provide signed informed consent.

The study is reported following the Transparent Reporting of Evaluations with Nonrandomized Designs (TREND) statement (2004).

Participating physicians received a training programme on palliative care specifically designed for the study, including guidance on how to implement a new consultation model designed for patients with palliative care needs in primary care. (Table 1) Subsequently, physicians were asked to identify patients in their lists of patients who had palliative care needs, based on a two-step process: first, all patients with a diagnosis of neoplasia, heart failure, chronic obstructive pulmonary disease, and chronic kidney disease were identified; then, by evaluating each patient clinical file, those who had advanced-stage disease were considered eligible. Eligible patients were randomly ordered using a computer software and contacted by telephone (in that same random order) to invite them to participate in the study. Each patient was required to provide signed informed consent. Those who accepted to take part received in-person consultations at each family physicians’ health centre, performed every 3 weeks for a total of 12 weeks (total of 5 consultations), according to the consultation model.

**Table 1.** Primary palliative care consultation model

	0 weeks	3 weeks	6 weeks	9 weeks	12 weeks
**Summarize clinical information (S)**					
Main diagnosis and others clinical problems	x				
Register chronic medication and SOS	x				
Vulnerabilities (financial, housing, nutrition, security, caregiver, literacy)	x	x	x	x	x
**Objective symptoms’ management (O)**					
Recording the result of the IPOS patient version – physical symptoms	x	x	x	x	x
Other symptoms	x	x	x	x	x
Adverse medication reactions	x	x	x	x	x
**Assessment and coding of chronic diseases and symptoms (A)**					
**Planning clinical approach (P)**					
Symptomatic treatment	x				
Review of the therapeutic record according to clinical relevance (e.g., description)	x	x	x	x	x
**(other) Problems/concerns (P)**	x	x	x	x	x

S, O, A, P – Portuguese Primary care registration form in electronic software. ‘S’ – Summarize clinical information; ‘O’ – Objective symptoms’ management, ‘A’ – Assessment and coding both chronic diseases and symptoms and ‘P’ – Planning clinical approach and (other) Problems/concerns.

We designed and powered the study for the primary outcome – patients’ physical symptoms’ burden, measured using the Integrated Palliative care Outcome Scale (IPOS) – patient version (Murtagh *et al.*, 2019). We calculated a sample size of 35 patients, considering two time points (at baseline and at 12 weeks), a power of 80%, a type I error of 5% and a medium effect size of 0.5 on physical symptoms’ burden (Cohen, 1992). To accommodate around 35% loss of patients at follow-up (Jordhøy MS *et al.*, 1999; Bouça-machado *et al.*, 2017; Perez-cruz *et al.*, 2018) we planned to recruit 53 patients. Secondary outcomes included emotional symptoms (IPOS), practical/communication issues (IPOS), and healthcare utilization. The latter included number medical consultations for acute disease in the primary healthcare unit, emergency department attendances, hospital admissions and referrals to hospital outpatient department, measured for the 12-week period prior the first consultation, during the 12-week period during consultations and for the 12-weeks after the last consultation.

Patient information was collected by each family physician using a pre-defined paper form including all the outcomes described above and also patient’s age, sex and main diagnosis. Healthcare utilization data were collected by the family physicians from clinical records. Each form was pseudonymized by assigning an ID code, so that only each family physician was able to identify which patient each form belonged to. The codebook was kept only by the physician.

Patient data were collected by each family physician using pre-defined paper forms. To ensure the reliability of the manually collected data and minimize potential entry errors, all completed forms underwent a thorough review for completeness and legibility. Data from these forms were entered by a single researcher.

This study reports on the secondary outcomes of a study that evaluated whether a complex intervention in primary care for patients with palliative care needs impacted their healthcare utilization.

We found a reduction in physical symptom burden with medium-large effect size and in emotional symptom burden with medium effect size, with no difference found for communication/practical issues (results reported elsewhere) (Seiça Cardoso *et al.*, 2024). We hereby report study results regarding healthcare utilization.

We first calculated medians and interquartile ranges to describe healthcare utilization variables across the three 12-week periods (before, during, and after the intervention). As all medians and interquartile ranges were 0.00 for all healthcare utilization variables at all three evaluation points, we subsequently present means and standard deviations to further describe these variables. To compare healthcare utilization across the 12-week periods, we primarily used Wilcoxon signed-rank tests due to the right-skewed and non-normally distributed nature of the data (Supplementary file 1). For completeness, paired-samples t-tests were also performed, which yielded similar results to the non-parametric tests (Supplementary file 2). All analyses were conducted in SPSS version 27, with *P* < 0.05 considered statistically significant.

## Results

Ten family physicians agreed to collaborate, and all received the training programme. After the training and before starting patients’ recruitment, one physician withdrew for personal reasons, leaving nine to recruit patients and deliver the consultation model. The 9 physicians (7 female) had a mean age of 33.8 y (SD 3.0) and a mean of 3.6 y (SD 3.2) of practice as a family physician. Three physicians referred they had short training in palliative care and one physician completed a one-month internship in a home palliative care team; the remaining had no training in palliative care.

We recruited the planned 53 patients, of which 18 were lost to follow-up (1 died, 9 due to clinical worsening, 3 dropped out, and 4 due to family physician sick leave) and 36 patients completed the intervention. These 36 patients had a mean age of 72.1 y (SD 12.9) and 52.8% were female. Fourteen had advanced-stage neoplasm, 12 had advanced congestive heart failure, 6 had advanced chronic kidney disease and 4 had advanced congestive obstructive pulmonary disease. Three patients died during the 12-weeks after the last consultation, so we analysed healthcare utilization for 33 patients.

The percentage of patients with no medical consultations for acute disease in the primary healthcare unit was 84.80% before the intervention, this remained stable during the intervention (84.80%) and then increased to 93.90% after the intervention. (Table 2) The percentage of patients with no emergency department attendances increased from 75.80% before the intervention to 81.80% during the intervention and to 90.9% after the intervention. The percentage of patients with no hospital admissions increased from 81.80% before the intervention to 90.90% during the intervention but decreased to 84.80% after the intervention (still higher than before the intervention). The percentage of patients with no referrals to hospital outpatient department increased from 84.80% before the intervention to 93.90% during the intervention and remained stable (93.90%) after the intervention.

**Table 2.** Patients’ health care utilization before, during and after the intervention

		*N* = 33								
		12 weeks before the intervention		During the 12-week intervention		12 weeks after the intervention				
		% (*n*)	Mean (sd)	%	Mean (sd)	%	Mean (sd)	*p* (b–d)	*p* (d–a)	*p* (b–a)
Medical acute consultation in primary healthcare unit	0 1 2	84.80 (28) 12.10 (4) 3.00 (1)	0.18 (0.47)	84.80 (28) 15.20 (5) 0.00 (0)	0.15 (0.36)	93.90 (31) 6.10 (2) 0.00 (0)	0.06 (0.24)	0.786	0.263	0.211
Emergency department attendances	0 1 2 3	75.80 (25) 15.20 (5) 9.10 (3) 0.00 (0)	0.33 (0.65)	81.80 (27) 15.20 (5) 3.00 (1) 0.0 (0)	0.24 (0.61)	90.90 (30) 6.10 (2) 0.00 (0) 3.00 (1)	0.15 (0.57)	0.414	0.083	0.110
Hospital admissions	0 1 2	81.80 (27) 15.20 (5) 3.00 (1)	0.21 (0.49)	90.90 (30) 9.10 (3) 0.00 (0)	0.09 (0.29)	84.80 (28) 15.20 (5) 0.00 (0)	0.15 (0.36)	0.211	0.423	0.535
Referrals to hospital outpatient department	01 2	84.80 (28) 15.20 (5) 0.00 (0)	0.15 (0.36)	93.90 (31) 3.00 (1) 3.00 (1)	0.09 (0.38)	93.90 (31) 3.00 (1) 3.00 (1)	0.09 (0.38)	0.423	1.000	0.488

(b–d) – comparison of the distribution of the healthcare utilization variables between the 12-week periods before the intervention and the 12-weeks during the intervention.

*p* (d–a) – *p* value comparing the distribution of the healthcare utilization variables between the 12-weeks during the intervention and the 12-weeks after the last consultation of each patient.

*p* (b–a) – comparison of the distribution of the healthcare utilization variables between the 12-week periods before the intervention and the 12-weeks after the last consultation of each patient.

sd – standard deviation.

We observed a decrease in the mean number of medical for acute disease in the primary healthcare unit (mean 0.18; SD 0.47) and the intervention period (mean 0.15; SD 0.36), with a further reduction during the post-intervention period (mean 0.06; SD 0.24). The mean number of emergency department attendances also decreased from the pre-intervention period (mean 0.33; SD 0.65) to the intervention period (mean 0.24; SD 0.61), with a further reduction in the post-intervention period (mean 0.15; SD 0.57). The mean number of hospital admissions fell from the pre-intervention period (mean 0.21; SD 0.49) to the intervention period (mean 0.09; SD 0.29), followed by a subsequent increase in the post-intervention period (mean 0.15; SD 0.36), although the mean remained lower than in the pre-intervention period. A reduction was also observed in the mean number of referrals to hospital outpatient department from the pre-intervention period (mean 0.15; SD 0.36) to the intervention period (mean 0.09; SD 0.38), which remained stable in the post-intervention period (mean 0.09; SD 0.38). Despite observed trends, none of these changes reached statistical significance. For medical acute consultations in the primary healthcare unit, the p-values were: 0.786 (*T*-test) and 0.782 (Wilcoxon) comparing before-during periods; 0.263 (*T*-test) and 0.257 (Wilcoxon) comparing during-after periods; and 0.211 (*T*-test) and 0.206 (Wilcoxon) comparing before-after periods. For emergency department attendances, the p-values were: 0.414 (*T*-test) and 0.405 (Wilcoxon) for before-during; 0.083 (*T*-test) and 0.083 (Wilcoxon) for during-after; and 0.110 (*T*-test) and 0.107 (Wilcoxon) for before-after. For hospital admissions, the p-values were: 0.211 (*T*-test) and 0.206 (Wilcoxon) for before-during; 0.423 (*T*-test) and 0.414 (Wilcoxon) for during-after; and 0.535 (*T*-test) and 0.527 (Wilcoxon) for before-after. Finally, for referrals to hospital outpatient department, the p-values were: 0.423 (*T*-test) and 0.414 (Wilcoxon) for before-during; 1.000 (*T*-test) and 1.000 (Wilcoxon) for during-after; and 0.488 (*T*-test) and 0.480 (Wilcoxon) for before-after (Table 2 and Supplementary File 2).

## Discussion

This pilot study, while not demonstrating statistically significant changes, provides crucial insights into the feasibility and potential of such interventions, and importantly, offers valuable data for informing future research, particularly in the context of sample size calculations for subsequent economic analyses of primary palliative care.

Based on our estimates, we would need 818 patients to show with 80% power and 0.05 confidence that the decreases we observed in the means for all measured services were statistically significant.

Notwithstanding, the impact on healthcare utilization was lower than what we expected and there may be several explanatory reasons. Firstly, the focus of the intervention was on improving clinical outcomes, not on preventing secondary care utilization. An additional intervention component could be introduced to help achieve this secondary outcome whenever appropriate. Secondly, healthcare utilization was low at baseline for all measured services; hence a reduction was not applicable to most patients and thus it was unlikely as a group. Thirdly, the results on clinical outcomes show the level of symptom burden was also generally low.

A longer length of intervention and follow-up would capture a longer trajectory of likely decline which may increase the potential for impact of the intervention on both clinical outcomes and healthcare utilization. This would pave the way to demonstrate cost-effectiveness.

## Conclusion

This pilot study provides crucial data for future research, particularly informing sample size calculations for economic analyses of primary palliative care. While we did not achieve a statistically significant reduction in healthcare utilization, we observed some tendencies towards its reduction. We propose that incorporating an additional intervention component specifically focused on preventing unnecessary secondary care utilization, alongside a longer intervention and follow-up period, could be effective strategies to enhance the potential for economic impact in future, larger-scale studies.

## Supporting information

Seiça Cardoso et al. supplementary material 1 Seiça Cardoso et al. supplementary material

Seiça Cardoso et al. supplementary material 2 Seiça Cardoso et al. supplementary material

## Data Availability

All data generated or analysed during this study are included in this published article [and its supplementary information files]. No new data were generated or analysed in support of this research.

## Supplementary material

The supplementary material for this article can be found at https://doi.org/10.1017/S1463423625100777.
