# Supplementary material for: Impact of a complex intervention in primary care for patients with palliative care needs in their healthcare utilization: a before-after study
Source: Prim Health Care Res Dev. 2026 Jan 30;27:e15. doi: 10.1017/S1463423625100777 (PMC12931984; doi:10.1017/S1463423625100777)
Supplement: Seiça Cardoso et al. supplementary material 1 — Seiça Cardoso et al. supplementary material [file S1463423625100777sup001.docx]

Supplementary file 1

Testing variables to verify normality.

|  | Shapiro-Wilk | |
| --- | --- | --- |
|  | Statistic | p |
| Medical acute consultations in primary care before | 0.446 | <0.001 |
| Medical acute consultations in primary care during | 0.565 | <0.001 |
| Medical acute consultations in primary care after | 0.493 | <0.001 |
| Emergency department attendances | 0.431 | <0.001 |
| Emergency department attendances | 0.431 | <0.001 |
| Emergency department attendances | 0.449 | <0.001 |
| Hospital admissions before | 0.328 | <0.001 |
| Hospital admissions during | 0.257 | <0.001 |
| Hospital admissions after | 0.259 | <0.001 |
| Referrals to hospital outpatient department before | 0.300 | <0.001 |
| Referrals to hospital outpatient department during | 0.431 | <0.001 |
| Referrals to hospital outpatient department after | 0.257 | <0.001 |
