# Supplementary material for: Impact of a complex intervention in primary care for patients with palliative care needs in their healthcare utilization: a before-after study
Source: Prim Health Care Res Dev. 2026 Jan 30;27:e15. doi: 10.1017/S1463423625100777 (PMC12931984; doi:10.1017/S1463423625100777)
Supplement: Seiça Cardoso et al. supplementary material 2 — Seiça Cardoso et al. supplementary material [file S1463423625100777sup002.docx]

Supplementary file 2

Related-Samples Wilcoxon Signed Rank Test results

|  | Z | p |
| --- | --- | --- |
| Medical acute consultations before-during | 25.000 | 0.782 |
| Medical acute consultations during-after | 8.000 | 0.257 |
| Medical acute consultations before-after | 21.000 | 0.206 |
| Emergency department attendances before-during | 20.000 | 0.405 |
| Emergency department attendances during-after | 0.000 | 0.083 |
| Emergency department attendances before-after | 29.000 | 0.107 |
| Hospital admissions before-during | 7.000 | 0.206 |
| Hospital admissions during-after | 14.000 | 0.414 |
| Hospital admissions before-after | 17.500 | 0.527 |
| Referrals to hospital outpatient department before-during | 7.000 | 0.414 |
| Referrals to hospital outpatient department during-after | 3.000 | 1.000 |
| Referrals to hospital outpatient department before-after | 10.000 | 0.480 |

Z –Related-Samples Wilcoxon Signed Rank Test
